# Supplementary material for: Loss-of-function mutations in Keratin 32 gene disrupt skin immune homeostasis in pityriasis rubra pilaris
Source: Nat Commun. 2024 Jul 24;15:6259. doi: 10.1038/s41467-024-50481-z (PMC11269665; doi:10.1038/s41467-024-50481-z)
Supplement: Supplementary file 1 — Supplementary Information [file 41467_2024_50481_MOESM1_ESM.pdf]

Loss-of-function mutations in Keratin 32 gene disrupt skin immune homeostasis in pityriasis rubra pilaris

Peidian Shi[#1,2], Wenjie Chen[#1,2], Xinxing Lyu[1,2], Zhenzhen Wang[1,2], Wenchao Li[1,2], Fengming Jia[1,2], Chunzhi Zheng[1,2], Tingting Liu[1,2], Chuan Wang[1,2], Yuan Zhang[1,2], Zihao Mi[1,2], Yonghu Sun[1,2], Xuechao Chen[1,2], Shengli Chen[1,2], Guizhi Zhou[1,2], Yongxia Liu[1,2], Yingjie Lin[1,2], Fuxiang Bai[1,2], Qing Sun[3], Monday O. Ogese[4], Qiang Yu[5], Jianjun Liu[5], Hong Liu[1,2,6*], Furen Zhang[1,2,6,7*]

[1]Hospital for Skin Diseases, Shandong First Medical University, Jinan, Shandong, China

[2]Shandong Provincial Institute of Dermatology and Venereology, Shandong Academy of Medical Sciences, Jinan, Shandong, China

[3]Department of Dermatology, Qilu Hospital, Shandong University, Jinan, Shandong, China

[4]Department of Pharmacology and Therapeutics, University of Liverpool, Liverpool, United Kingdom

[5]Genome Institute of Singapore, Singapore

[6]School of Public Health, Shandong First Medical University and Shandong Academy of Medical Sciences, Jinan, Shandong, China

[7]Shandong University of Traditional Chinese Medicine, Jinan, Shandong, China

[#] These two authors contributed equally to this work.

***Correspondence to:** Furen Zhang: zhangfuren@hotmail.com**;** Hong Liu. Email: hongyue2519@hotmail.com and Telephone number, 86-0531-87298870. Fax number, 86-0531-87984734.

Supplementary information

Supplementary Figure 1. KRT32 expression in human tissues and specifically in skin cells.

Supplementary Figure 2. Construction of Ker-CT cell lines with KRT32 overexpression and knockdown.

Supplementary Figure 3. Expression of inflammatory cytokines in the serum and skin of PRP patients.

Supplementary Figure 4. Immunohistochemical staining for detecting TNF, IL-1β, IL-6 and IL-8 in the epidermis of PRP patients.

Supplementary Figure 5. KRT32 physically interacts with NEMO.

Supplementary Figure 6. Double immunofluorescent staining of NEMO and KRT32 of the lesioned skins from PRP patients with/without *KRT32* mutations

Supplementary Figure 7. Generation of *Krt32* KO mice by CRISPR-Cas9.

Supplementary Figure 8 (Related to Figure 8). The skin phenotype in *Krt32* KO mice induced by TNF.

Supplementary Figure 9. SEM observation of hair and nail surfaces in *Krt32* wild-type and KO mice.

Supplementary Figure 10. The ultrastructure of keratinocytes in a model of PRP-like dermatitis *Krt32*$^{(-/-)}$ mice observed by electron microscopy.

Supplementary Figure 11. Illustration of the mechanism of PRP caused by pathogenic mutations of *KRT3*2.

Supplementary Table 1. Summary of PRP patients with damaging mutations.

Supplementary Table 2. Clinical characteristics of 102 PRP patients.

Supplementary Table 3. Primers used in the site directed mutagenesis of KRT32.

Supplementary Table 4. Primers used for RT-qPCR amplification.

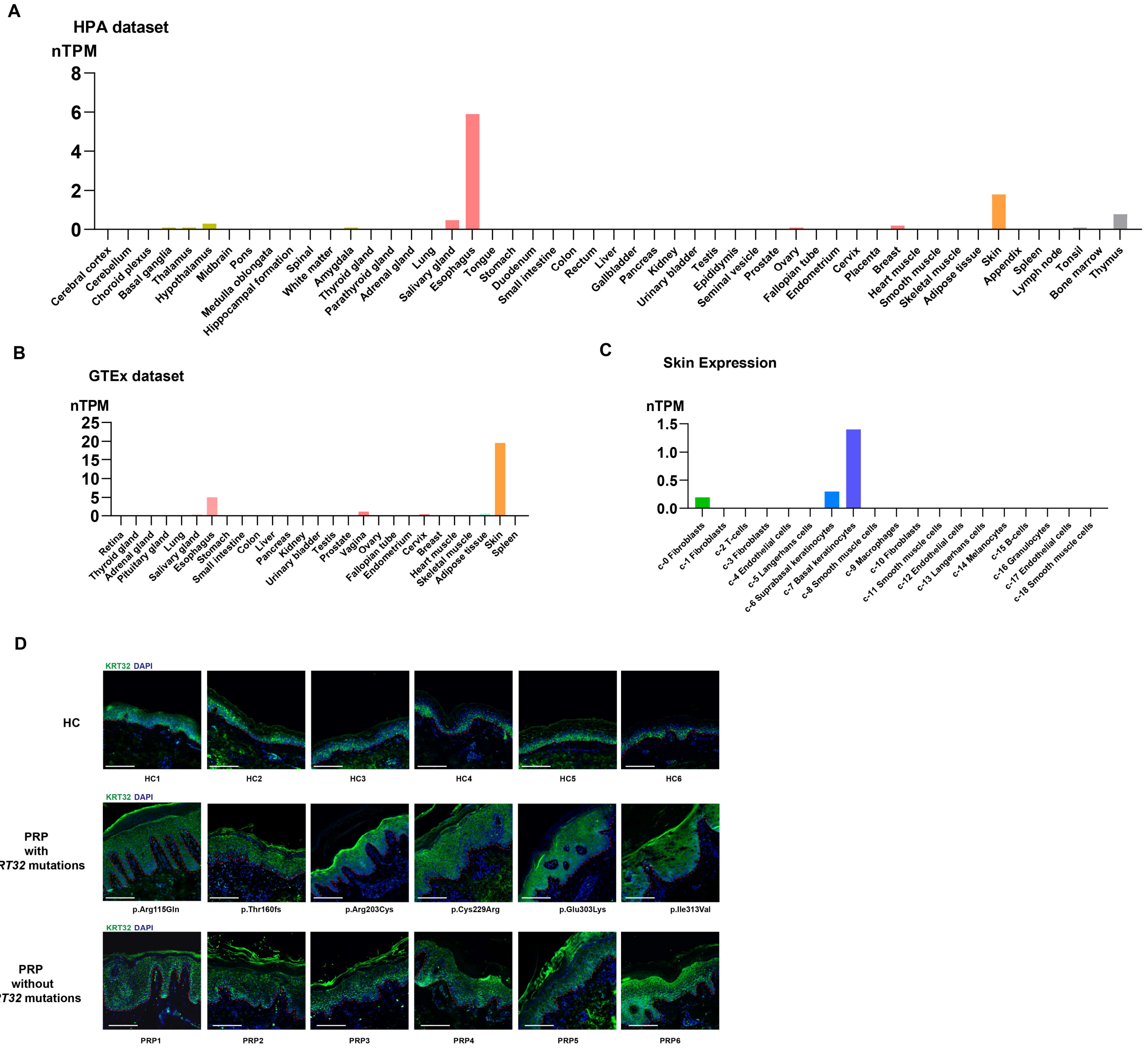

Supplementary Figure 1. KRT32 expression in human tissues and specifically in skin cells.

(A-B) Tissue-specific expression of KRT32 based on the two independent consortia of Human Protein Atlas (HPA) and Genotype-Tissue Expression (GTEx). (C) Cell type-specific expression of KRT32 in human skin. (D) Immunohistochemical analysis of KRT32 expression in skin tissues obtained from PRP patients with *KRT32* mutations, PRP patients without *KRT32* mutations (n = 6), and healthy controls (n = 6). Age, gender, and skin sampling site matching were ensured between healthy individuals and patients. Scale bar = 150 μm. The red dotted line denotes the epidermal-dermal boundary.

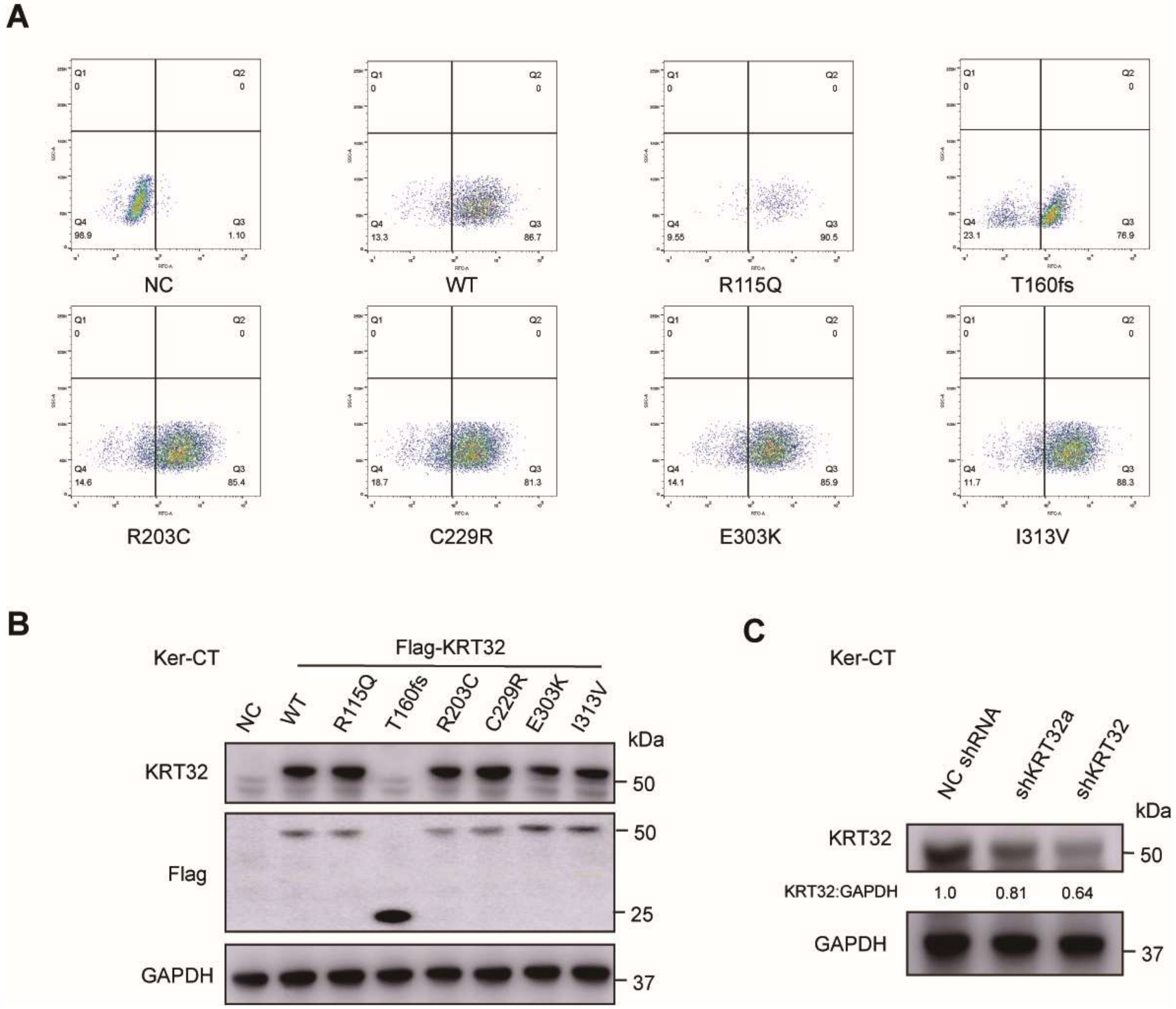

Supplementary Figure 2. Construction of Ker-CT cell lines with KRT32 overexpression and knockdown.

(A, B) The efficiency of KRT32 wildtype and mutations overexpression in Ker-CT cells was validated by flow cytometric and Western blot analysis using anti-Flag monoclonal antibody. (C) The efficiency of KRT32 knockdown in Ker-CT cells was verified by Western blot analysis. Two different shRNAs (shKRT32a and shKRT32) were used here. One representative experiment from two independent experiments with similar results is shown in B and C. Source data are provided as Source Data file.

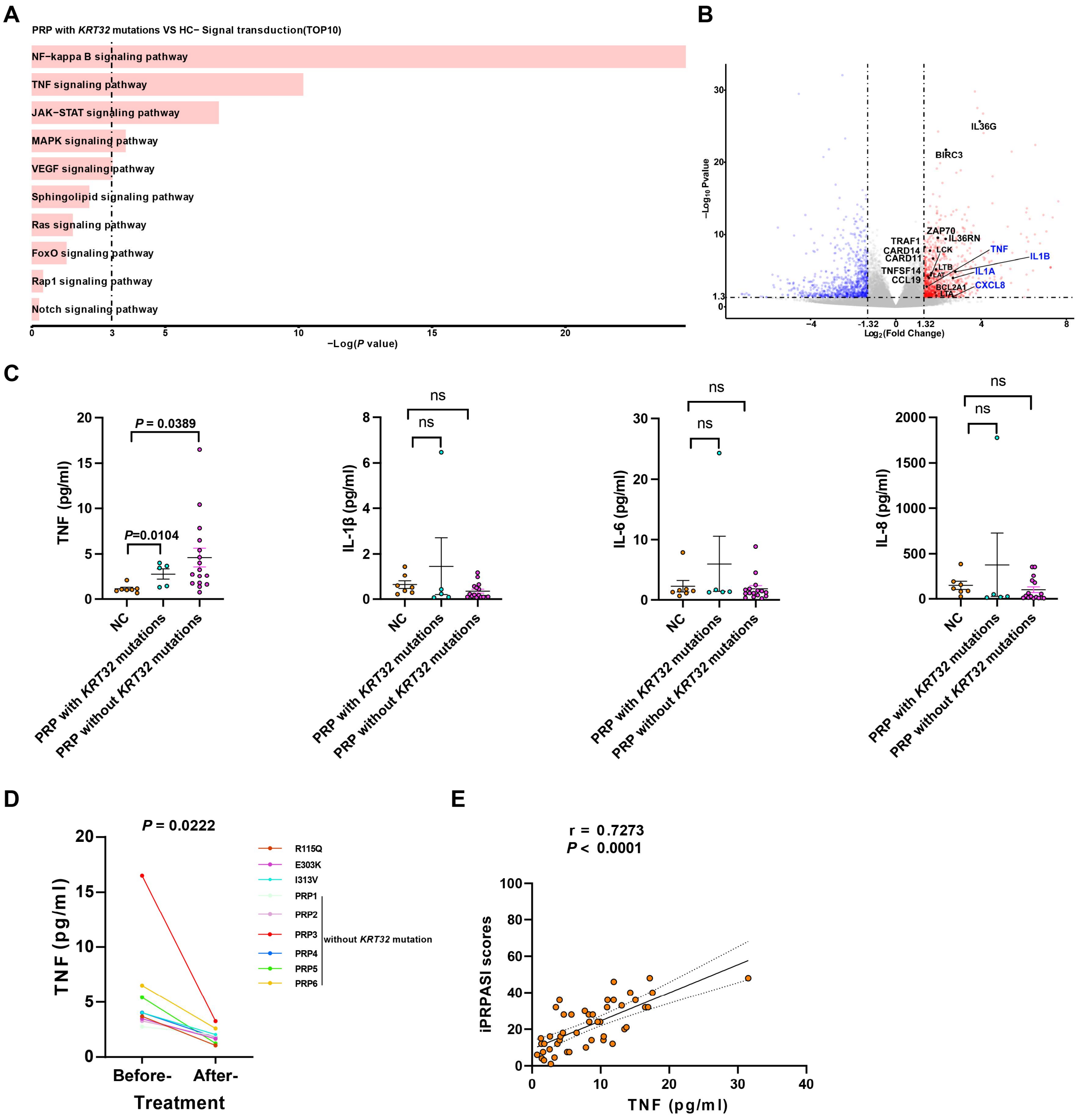

Supplementary Figure 3. Expression of inflammatory cytokines in the serum and skin of PRP patients.

(A) Selected KEGG signal transduction pathways were identified for significant DEGs (P <0.05 and $\log_2$FC >0.585) through RNA sequencing of FFPE samples from six PRP patients with *KRT32* mutations, along with six healthy controls and patient-matched normal skin tissues. The statistical test was hypergeometric test, and the level of significance was set at a two-sided $P < 0.05$ without multiple comparisons. (B) Volcano plot illustrating significant DEGs between PRP patients with *KRT32* mutations and healthy controls. Colored points represent $P <$ 0.05, which means −log (P) > 1.30, dashed line), with red indicating upregulated genes ($\log_2$FC > 1.32) and blue indicating downregulated genes ($\log_2$FC < -1.32). Selected NF-κB pathway-associated genes are highlighted in black. FC denotes fold change. The statistical test was Wald test and the level of significance was set at a two-sided $P < 0.05$ without multiple comparisons. (C) The secretion of inflammatory cytokines in the serum of patients with PRP was measured by the MSD method. A two-sided unpaired Student's t-test was used for comparative analysis between PRP patients with *KRT32*

mutations (n = 5; individuals 1, 2, 3, 5, and 6), without *KRT32* mutation (n = 16) and healthy controls (n = 7) with a significant *P* value ($P < 0.05$; ns, not significant). (D) The secretion of TNF in the serum of PRP patients with and without *KRT32* mutations was measured before treatment and healed (n = 9). *P* value was calculated using a two-sided unpaired Student's t test. (E) Correlation between serum TNF levels with PRP area and severity index (iPRPASI) scores in the study group (n = 47) were analyzed by Pearson correlation analysis (Pearson's r = 0.7273, $P < 0.0001$). Source data are provided as Source Data file.

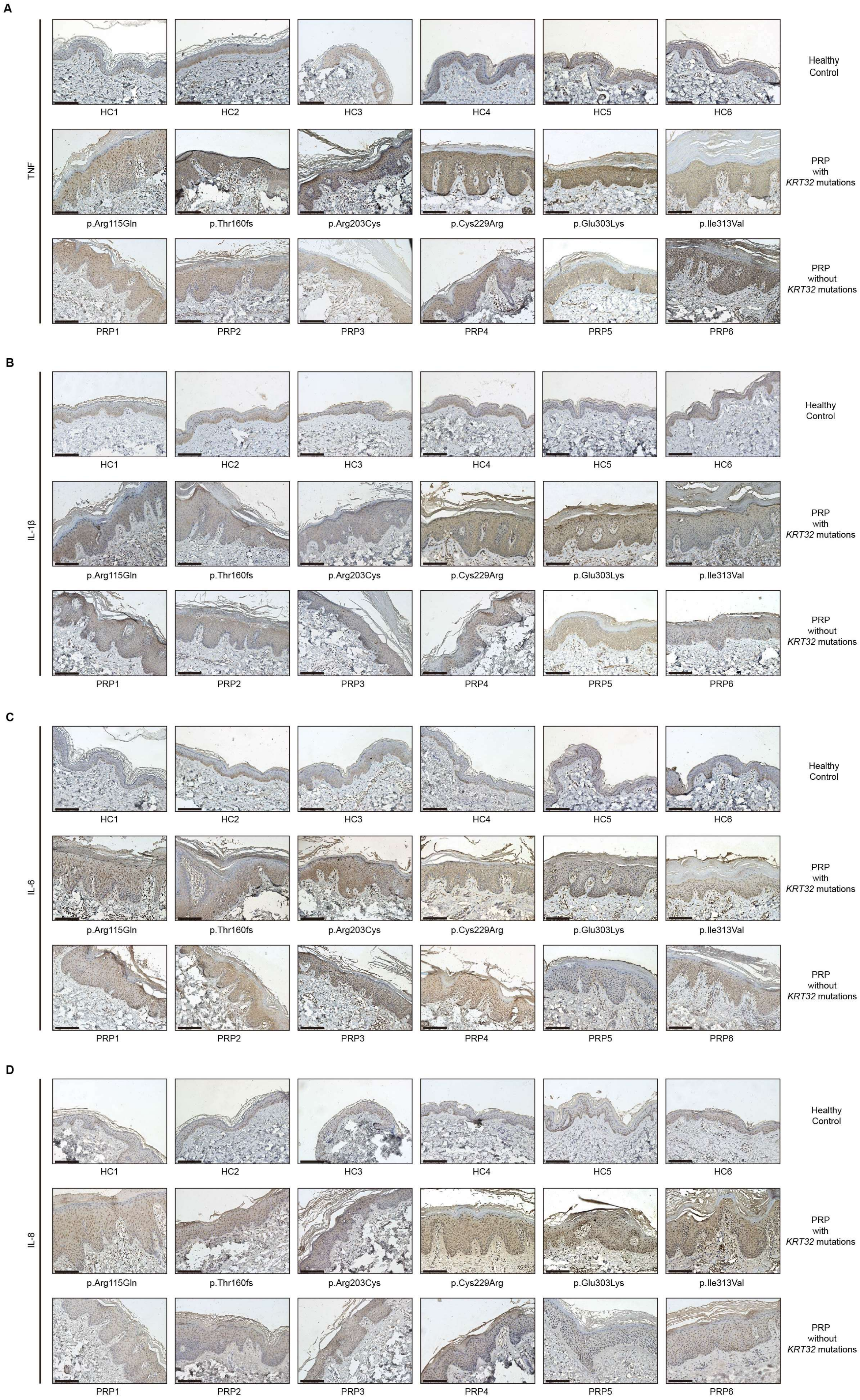
A
TNF
HC1
HC2
HC3
HC4
HC5
HC6
Healthy Control
p.Arg115Gln
p.Thr160fs
p.Arg203Cys
p.Cys229Arg
p.Glu303Lys
p.Ile313Val
PRP with KRT32 mutations
PRP1
PRP2
PRP3
PRP4
PRP5
PRP6
PRP without KRT32 mutations
B
IL-1β
HC1
HC2
HC3
HC4
HC5
HC6
Healthy Control
p.Arg115Gln
p.Thr160fs
p.Arg203Cys
p.Cys229Arg
p.Glu303Lys
p.Ile313Val
PRP with KRT32 mutations
PRP1
PRP2
PRP3
PRP4
PRP5
PRP6
PRP without KRT32 mutations
C
IL-6
HC1
HC2
HC3
HC4
HC5
HC6
Healthy Control
p.Arg115Gln
p.Thr160fs
p.Arg203Cys
p.Cys229Arg
p.Glu303Lys
p.Ile313Val
PRP with KRT32 mutations
PRP1
PRP2
PRP3
PRP4
PRP5
PRP6
PRP without KRT32 mutations
D
IL-8
HC1
HC2
HC3
HC4
HC5
HC6
Healthy Control
p.Arg115Gln
p.Thr160fs
p.Arg203Cys
p.Cys229Arg
p.Glu303Lys
p.Ile313Val
PRP with KRT32 mutations
PRP1
PRP2
PRP3
PRP4
PRP5
PRP6
PRP without KRT32 mutations

Supplementary Figure 4. Immunohistochemical staining for detecting TNF, IL-1β, IL-6 and IL-8 in the epidermis of PRP patients.

Immunohistochemical staining was performed to assess the expression levels of TNF (A), IL-1β (B), IL-6 (C), and IL-8 (D) in skin biopsy sections obtained from different groups. Representative sections from healthy controls (n = 6) and patients with *KRT32* mutations were included, along with the patients without *KRT32* mutations (n = 6). Scale bar = 150 μm.

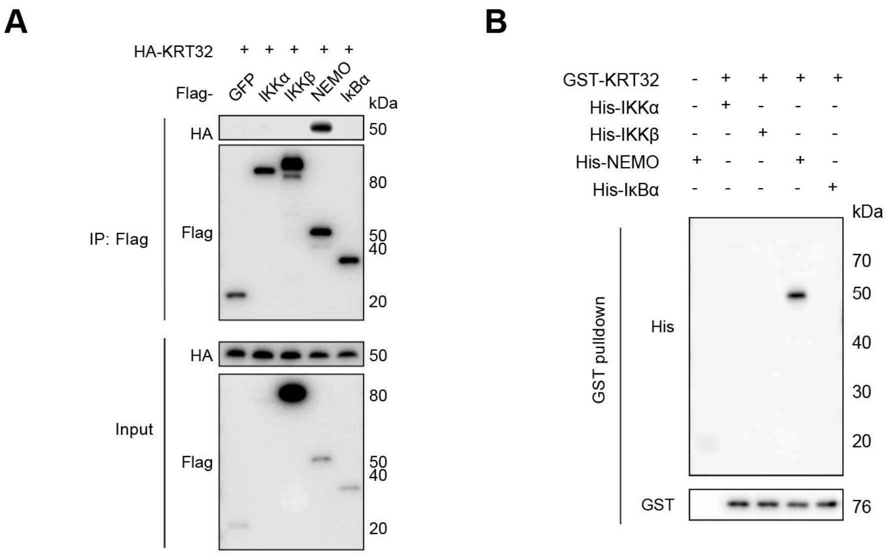

Supplementary Figure 5. KRT32 physically interacts with NEMO.

(A) HEK-293T cells were co-transfected with HA-KRT32 and the key adaptor protein of the NF-κB signaling pathway (Flag-IKKα, Flag-IKKβ, Flag-NEMO, Flag-IκBα, or control plasmids (Flag-GFP)). After 24 h, cell lysates were prepared, immunoprecipitated with anti-Flag antibodies, and subjected to immunoblotting assay for coprecipitated HA-tagged proteins. (B) The interaction of KRT32 and the adaptor proteins of NF-κB were analyzed by *in vitro* pull-down assay. E. coli extracts containing GST or GST-KRT32 proteins were incubated with E. coli extracts containing His-IKKα, His-IKKβ, His-NEMO, or His-IκBα and glutathione-Sepharose beads to pull down GST complexes. One representative experiment from three independent experiments with similar results is shown in A and B. Source data are provided as Source Data file.

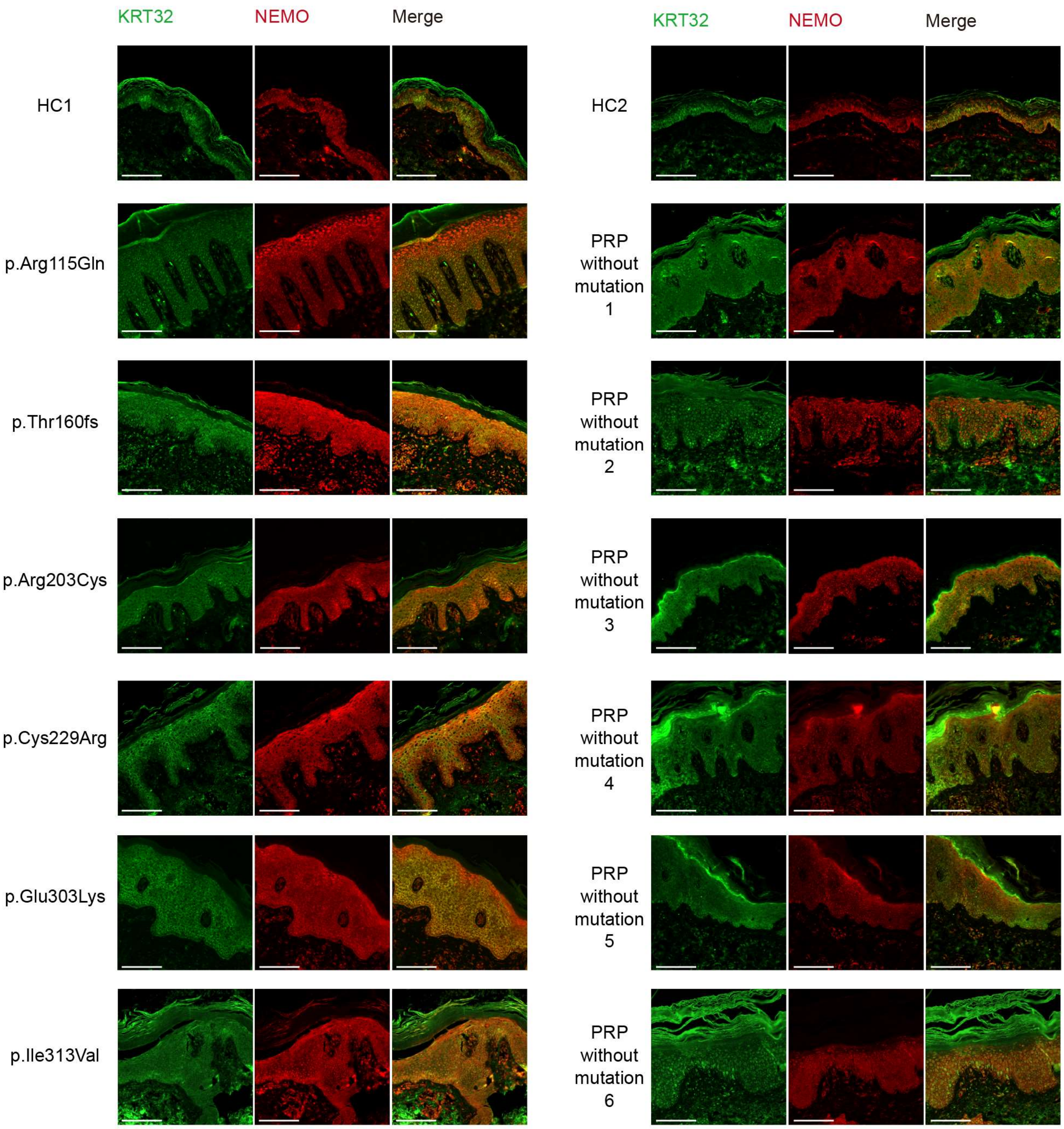

Supplementary Figure 6. Double immunofluorescent staining of NEMO and KRT32 of the lesioned skins from PRP patients with/without *KRT32* mutations

Skin tissue sections obtained from healthy controls and PRP patients with and without *KRT32* mutations were immunostained to detect NEMO and KRT32. Scale bars represent 150 μm. Representative immunohistochemical images of NEMO and KRT32 staining from two healthy individuals and six cases of PRP with and without *KRT32* mutations are present.

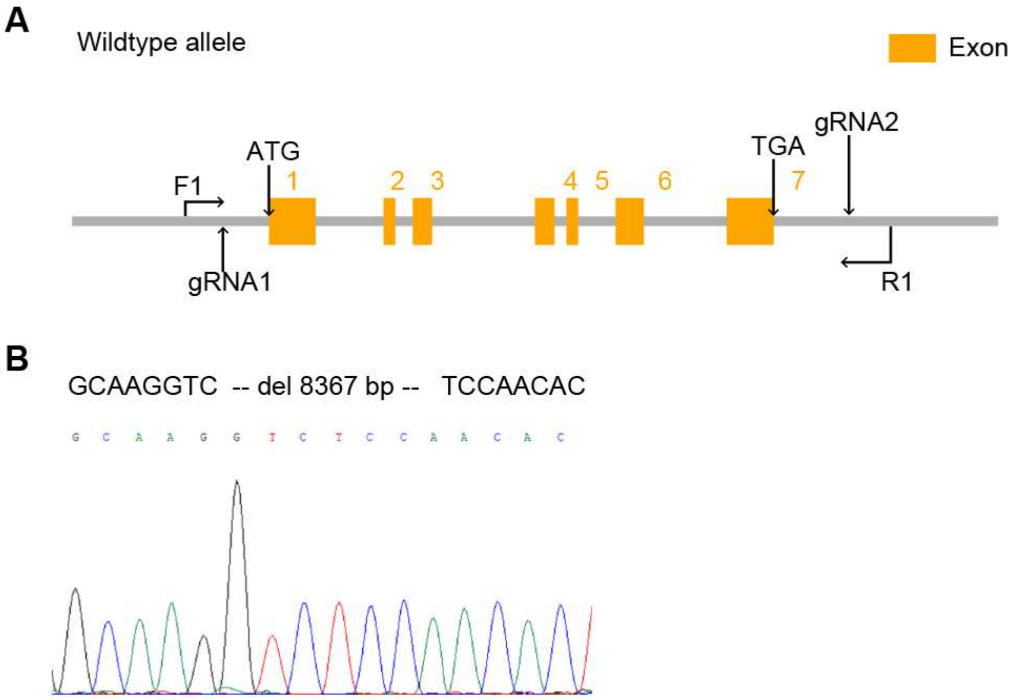

Supplementary Figure 7. Generation of *Krt32* KO mice by CRISPR-Cas9.

(A) Schematic of the strategy to construct of *Krt32* KO mice. gRNA1 and gRNA2 are the target regions of *Krt32* gene on the chromosome. F1 and R1 regions were used to design primers for genotyping. (B) The *Krt32* KO mice were validated with Sanger sequencing.

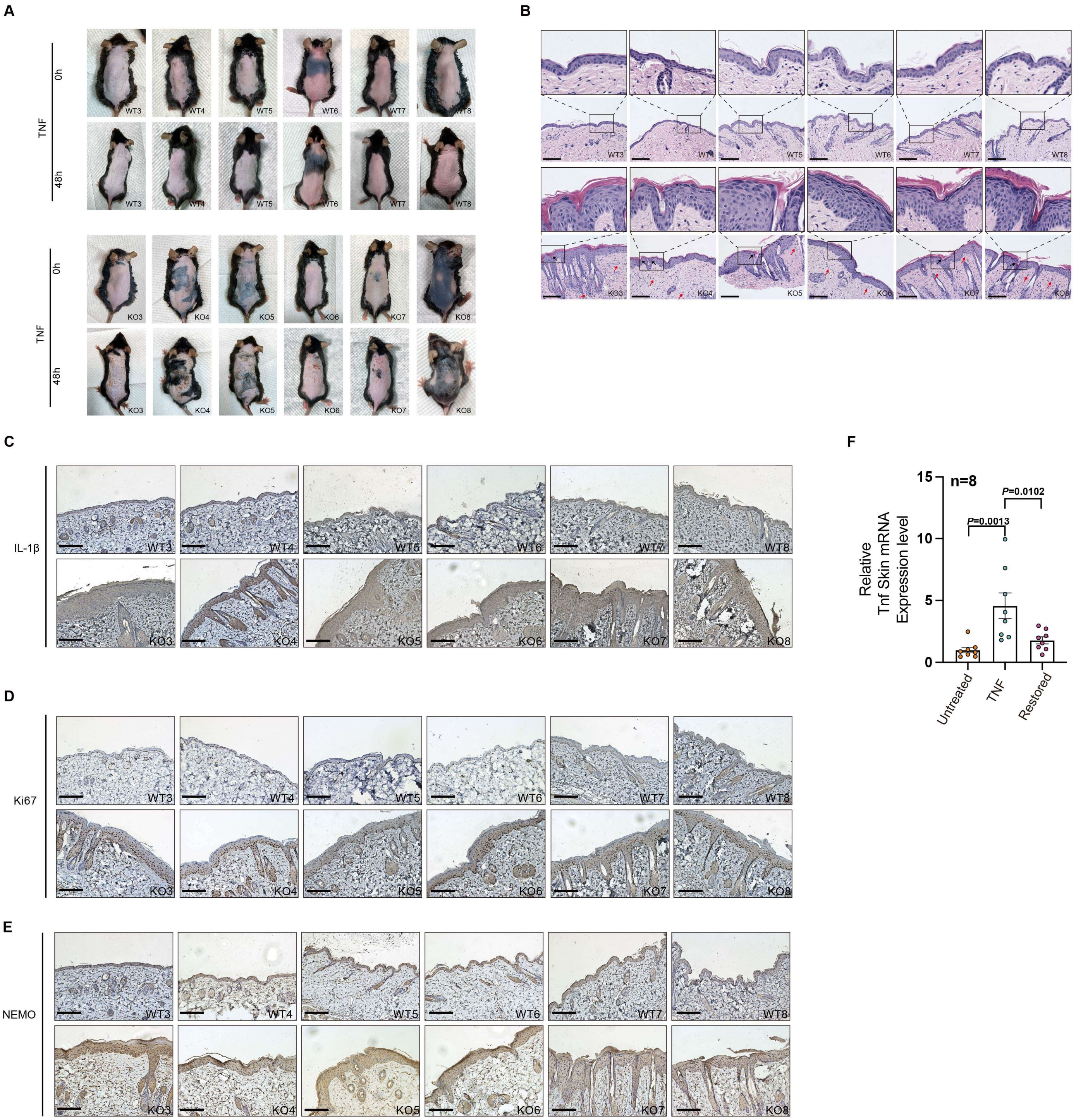

Supplementary Figure 8 (Related to Figure 8). The skin phenotype in *Krt32* KO mice induced by TNF.

(A-E). Skin phenotypes of the other 6 groups out of 8 groups of *Krt32* WT/KO mice, including the photographs of the dorsal skin (for Figure 8B), H&E staining (for Figure 8C), IL-1β (for Figure 8H), Ki67 (for Figure 8I), NEMO (for Figure 8E). (F) The *Tnf* transcription in the skin of *Krt32* KO mice with non-treatment, treatment and restoration from TNF treatment (n = 8 mice/group). Data are means ± SEM, and *P* value was calculated using a two-sided unpaired Student's t test. Source data are provided as Source Data file.

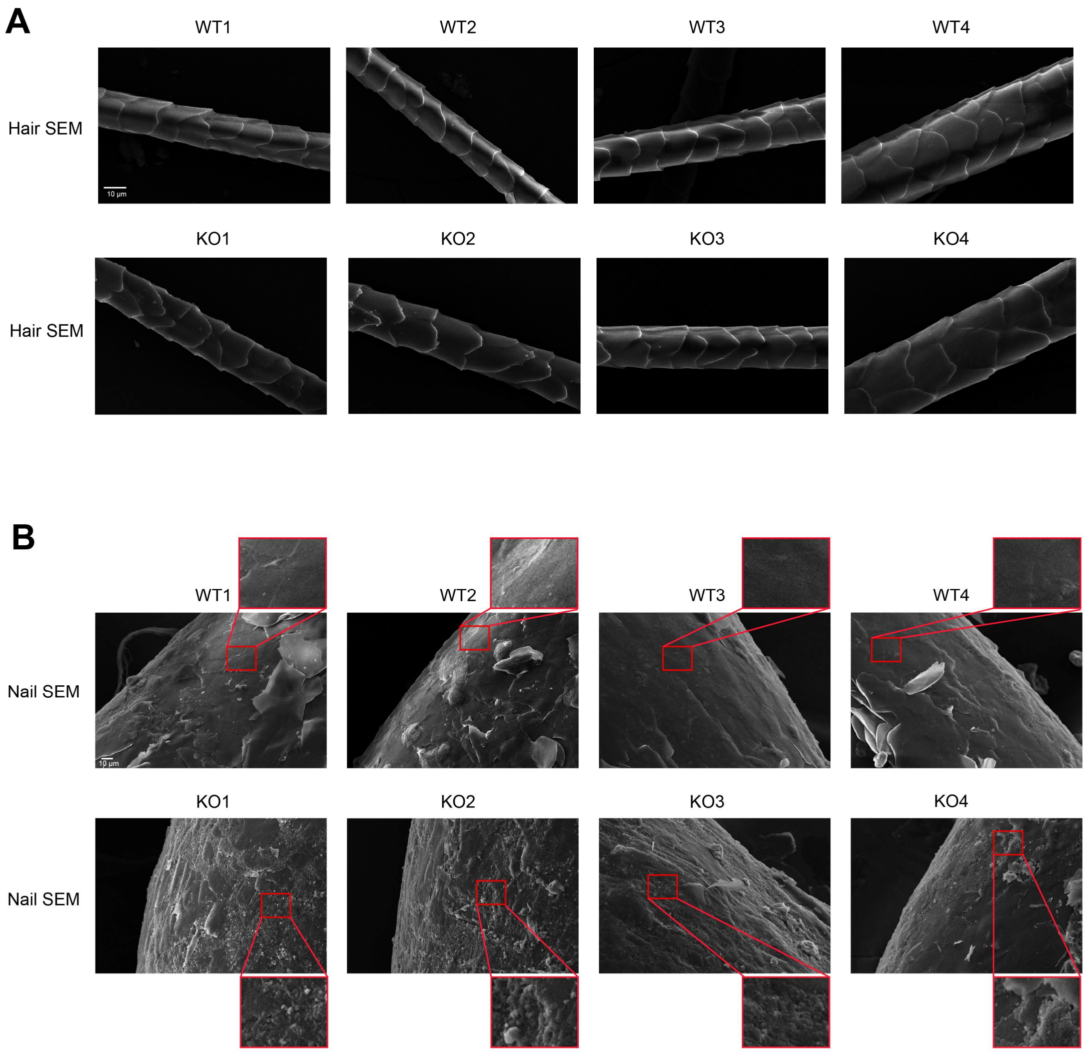

Supplementary Figure 9. SEM observation of hair and nail surfaces in *Krt32* wild-type and KO mice.

Hair samples from the backs (A) and nail samples from the hind limbs (B) of *Krt32* wild-type and knockout (KO) mice treated with TNF (n = 4 mice/group) were observed using scanning electron microscopy (SEM). Scale bar = 10 µm. Boxed areas are amplified in inserts to indicate roughness on the surface of nails.

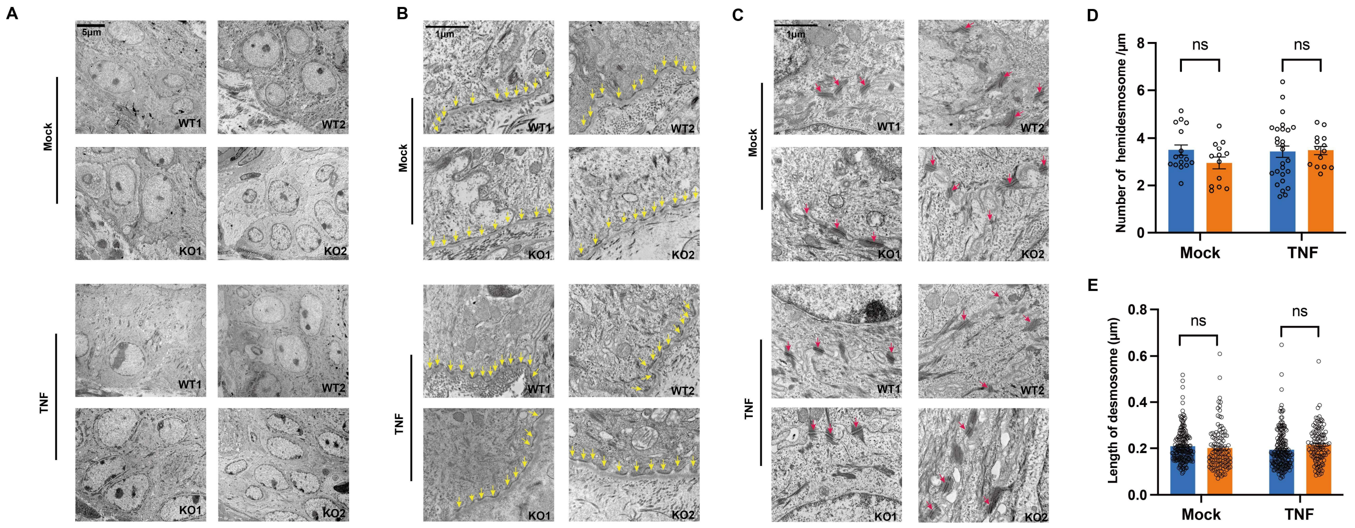

Supplementary Figure 10. The ultrastructure of keratinocytes in a model of PRP-like dermatitis *Krt32*$^{(-/-)}$ mice observed by electron microscopy.

(A) Representative electron microscopy images of keratinocyte cell phenotypes in *Krt32*$^{(-/-)}$ and wildtype mice observed with and without TNF treatment by. Scale bar = 5 µm. (B, C) Representative images of hemidesmosomes and desmosomes in keratinocytes from the PRP mouse model. Scale bar = 1 µm. Hemidesmosomes (arrows in yellow); Desmosomes (arrows in red). The process of capturing these images was repeated for four mice per group (n = 4 mice/group), yielding consistent results in A, B and C. (D) The number of hemidesmosomes per µm. At least 13 fields from different mice were quantified (n = 4 mice/group). (E) The length of individual desmosome, > 100 desmosomes was quantified from different mice (n = 4 mice/group). Data are means ± SEM, and *P* value was calculated using a two-sided unpaired Student's t test in D and E (ns; no significant difference). Source data are provided as Source Data file.

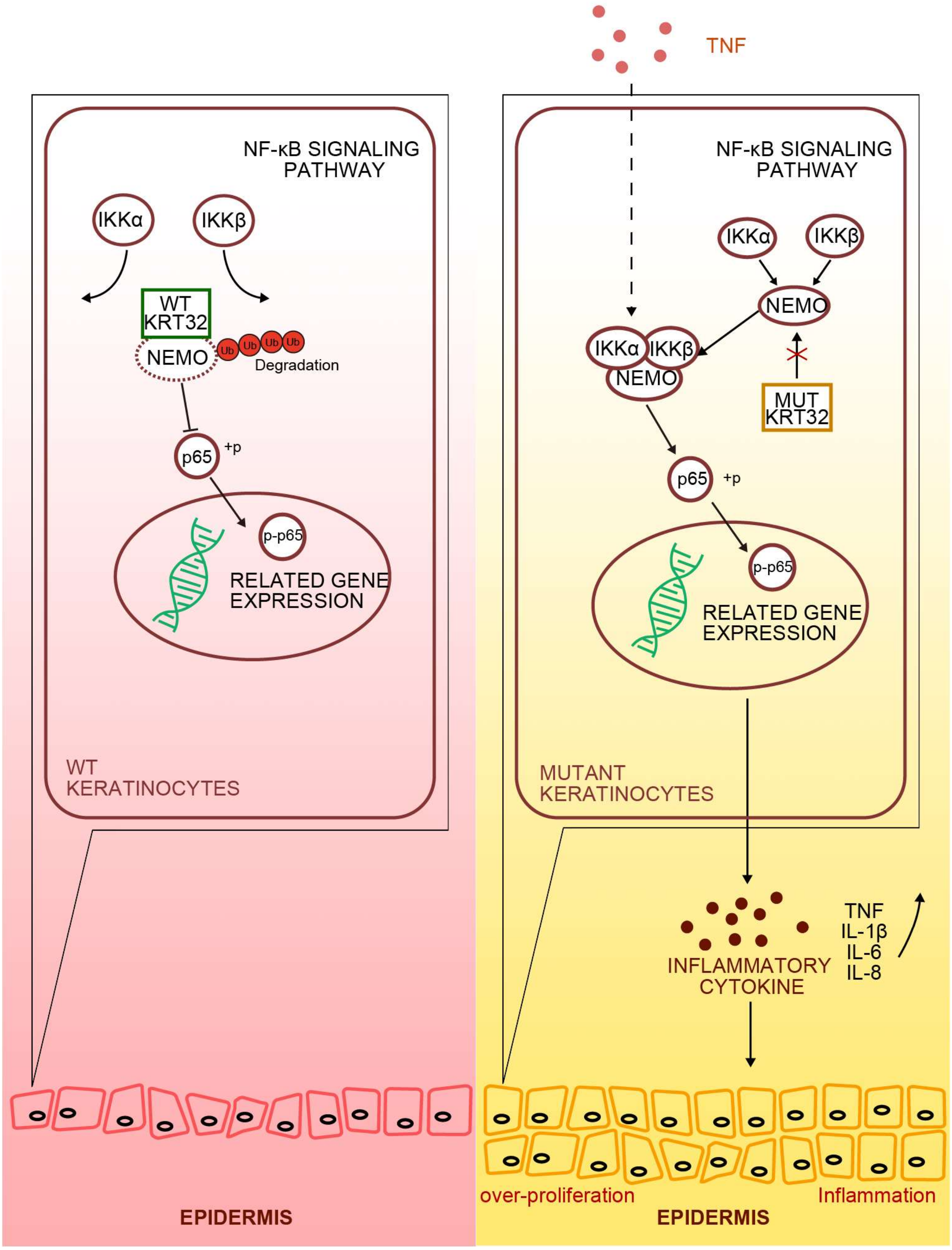

Supplementary Figure 11. Illustration of the mechanism of PRP caused by pathogenic mutations of *KRT32*.

The diagram illustrates the process of KRT32-based regulation of skin inflammation in PRP. The KRT32 interacts with NEMO, promotes its degradation through K48-linked polyubiquitination, and impedes the formation of IKK complex. Thus, a dual regulation of KRT32 helps to maintain skin immune responses balance. However, loss-of-function mutations in KRT32 among PRP patients result in NEMO-mediated NF-κB inflammatory axis hyperactivation, resulting in excessive secretion of inflammatory cytokines and ultimately causing skin inflammation and excessive keratinocyte proliferation in PRP.

Supplementary Table 1. Summary of PRP patients with damaging mutations.

Gene	Individuals No.	Gender	PRP subtype	Age	Age at onset (y)	Disease duration	AAChange	Genotype	Family history
KRT32	1	Male	II	53	49	4y	c.344G>A(p.Arg115Gln)	HET	negative
	2	Male	I	76	76	1m	c.477_478del(p.Thr160fs)	HET	negative
	3	Female	I	41	41	2m	c.607C>T(p.Arg203Cys)	HET	negative
	4	Female	II	36	19	18y	c.685T>C(p.Cys229Arg)	HET	negative
	5	Male	I	67	67	6m	c.907G>A(p.Glu303Lys)	HET	negative
	6	Female	I	54	54	2m	c.937A>G(p.Ile313Val)	HET	negative
CARD14	7	Male	I	42	41	1.3m	c.1799 C>T(p.Pro600Leu)	HET	negative

Supplementary Table 2. Clinical characteristics of 102 PRP patients.

Characteristic	Value. (%)
Sex	
Male	66 (64.7%)
Female	36 (35.3%)
Age(yr)	
Range	1-89
Mean	50.8
Age at on set	
Range	1-89
Mean	50.3
Red plaques	100 (98.4%)
Follicular Papules	62 (60.8%)
Nail involvement	24 (23.5%)
Palmoplantar keratoderma (PPK)	71 (69.6%)
Pruritus	89 (87.3%)
Ectropion	9 (8.8%)
Photosensitivity	5 (4.9%)
Hair loss	4 (3.9%)
Joint pain	5 (4.9%)
Family History	2 (2.0%, psoriasis)

Supplementary Table 3. Primers used in the site directed mutagenesis of KRT32.

Mutation	Primer pairs
R115Q	Forward: 5'- GGTGGAGGCCGACATCAATGGCCTGTGCAGGATCCTGGATGATCTCACTCTGT-3'
	Reverse: 5'- ACAGAGTGAGATCATCCAGGATCCTGCACAGGCCATTGATGTCGGCCTCCACC-3'
T160fs	Forward: 5'-GAGGGGTCACAGGGATGCCACCCGTCAATGTTCACAACCATCCTGGCATTCTCTGCCTTGGCACAGAATCTTCTGCTGGAGCTCCTC-3'
	Reverse: 5'- AGATCTGATATCGGTACCAGTCGACTCTAGAGGATCCATGACATCCTCCTGCTGTGTCACCAA-3'
R203C	Forward: 5'- CTGGCCAGCTACCTGACGAGGGTGCAGCAGCTGGAGCAGGAGAATGCGGA-3'
	Reverse: 5'- TCCGCATTCTCCTGCTCCAGCTGCTGCACCCTCGTCAGGTAGCTGGCCAG-3'
C229R	Forward: 5'- CCTCCTCATGGTTCTTTTTGAGGCGCATCAGCTCCTCCTTCAGGGACTCA-3'
	Reverse: 5'- TGAGTCCCTGAAGGAGGAGCTGATGCGCCTCAAAAAGAACCATGAGGAGG-3'
E303K	Forward: 5'- TAACCAACAGGTGGCCACAAGCTCTAAGCAGCTTCAGAACTACCAGTCAGA-3'
	Reverse: 5'- TCTGACTGGTAGTTCTGAAGCTGCTTAGAGCTTGTGGCCACCTGTTGGTTA-3'
I313V	Forward: 5'- GCTTCAGAACTACCAGTCAGACATCGTTGACCTGAGACGCACGGTCAACAC-3'
	Reverse: 5'- GTGTTGACCGTGCGTCTCAGGTCAACGATGTCTGACTGGTAGTTCTGAAGC-3'
R32Q	Forward: 5'- TCCAGTTCCTGCCGGGCAGCCAGTGGCATCTCCGGCTCCAT-3'
	Reverse: 5'- GATGCCACTGGCTGCCCGGCAGGAACTGGATAGATAGGTTT-3'
C51G	Forward: 5'- GGCGTGAACTGCCAGCCTGAGCTGTGCCTGGGCTATGTCTG-3'
	Reverse: 5'- CAGGCACAGCTCAGGCTGGCAGTTCACGCCGCTGGAACAGA-3'
Q72R	Forward: 5'- CTCCTGACTACCATTCTCATTTCAGGACCATTGAGGAGCTC-3'
	Reverse: 5'- TGGTCCTGAAATGAGAATGGTAGTCAGGAGTCATGGTGAGC-3'
Q144H	Forward: 5'- CCTGCCTTCGGTCGGCCTGCCCACCACCTTCCGGCCAGCCA-3'
	Reverse: 5'- AAGGTGGTGGGCAGGCCGACCGAAGGCAGGCATGCCATGGG-3'
I171T	Forward: 5'- ATGGTTGTGAACACTGATAATGCCAAACTGGCTGCCGATGA-3'
	Reverse: 5'- CAGTTTGGCATTATCAGTGTTCACAACCATCCTGGCATTCT-3'
V195L	Forward: 5'- GCCCAGGTTGAGTACCTGAAGGAGGAGCTGATGTGCCTCAA-3'
	Reverse: 5'- CAGCTCCTCCTTCAGGTACTCAACCTGGGCCTCCAGGTCAG-3'
S222Y	Forward: 5'- CATGCGGCAGCTGTTGGAGGCCGACATCAATGGCCTGCGCA-3'
	Reverse: 5'- TTGATGTCGGCCTCCAACAGCTGCCGCATGGCCAGCTCTGC-3'
R280H	Forward: 5'- GTGGAGGCCAACCACAGGGACGTGGAGGAATGGTTCAATAT-3'
	Reverse: 5'- TTCCTCCACGTCCCTGTGGTTGGCCTCCACCATGGCCTCGT-3'
R369Q	Forward: 5'- AGCCTGCTGGAGAGCGAGGACTGCAAGCTGCCCTGTAACCC-3'
	Reverse: 5'- CAGCTTGCAGTCCTCGCTCTCCAGCAGGCTCCGGTACGTGT-3'
D371E	Forward: 5'- GGCGAGATCAACATGTACCGGAGCCTGCTGGAGAACGAGGA-3'
	Reverse: 5'- CAGCAGGCTCCGGTACATGTTGATCTCGCCCTCCAGCCGGG-3'
T395M	Forward: 5'- AGATCCGGGCTGAGCTGGAGCGGCAGAACCAGGAGTACCAG-3'
	Reverse: 5'- GGTTCTGCCGCTCCAGCTCAGCCCGGATCTCAGCCAGCTGG-3'
N402S	Forward: 5'- CTCCCCATGCGTGACCCGCACCGTCTGTGTGCCACGCACTG-3'
	Reverse: 5'- ACACAGACGGTGCGGGTCACGCATGGGGAGGGCACACAGGT-3'
V426M	Forward: 5'- CCCATGCGTGCCCTGCACCGTCTGTGTGCCACGCACTGTTG-3'

	Reverse: 5'- GGCACACAGACGGTGCAGGGCACGCATGGGGAGGGCACACA-3'
P427T	Forward: 5'- CTGGCTGAGATCCAGGCTGACCTGGAGCGGCAGAACCAGGA-3'
	Reverse: 5'- CCGCTCCAGGTCAGCCTGGATCTCAGCCAGCTGGGCCTCAA-3'
R428C	Forward: 5'- GCCCTCCCCATGCATGCCCCGCACCGTCTGTGTGCCACGCA-3'
	Reverse: 5'- CAGACGGTGCGGGGCATGCATGGGGAGGGCACACAGGTGGT-3'

Supplementary Table 4. Primers used for RT-qPCR amplification.

Genes	Species	**Primer pairs**
IKBKG	Human	Forward: 5'- AGCACCTGAAGAGATGCCAGCA -3'
		Reverse: 5'- AGCCTGGCATTCCTTAGTGGCA -3'
ACTB	Human	Forward: 5'- GCCGGGACCTGACTGACTAC-3'
		Reverse: 5'- CGGATGTCCACGTCACACTT-3'
Il1b	Mouse	Forward: 5'-TGGACCTTCCAGGATGAGGACA -3'
		Reverse: 5'-GTTCATCTCGGAGCCTGTAGTG -3'
Il6	Mouse	Forward: 5'-TACCACTTCACAAGTCGGAGGC-3'
		Reverse: 5'- CTGCAAGTGCATCATCGTTGTTC-3'
Tnf	Mouse	Forward: 5'-GGTGCCTATGTCTCAGCCTCTT-3'
		Reverse: 5'- GCCATAGAACTGATGAGAGGGAG-3'
Actb	Mouse	Forward: 5'- CATTGCTGACAGGATGCAGAAGG-3'
		Reverse: 5'- TGCTGGAAGGTGGACAGTGAGG-3'
