# Supplementary material for: Loss-of-function mutations in Keratin 32 gene disrupt skin immune homeostasis in pityriasis rubra pilaris
Source: Nat Commun. 2024 Jul 24;15:6259. doi: 10.1038/s41467-024-50481-z (PMC11269665; doi:10.1038/s41467-024-50481-z)
Supplement: Supplementary file 3 — Description of Additional Supplementary Data [file 41467_2024_50481_MOESM3_ESM.pdf]

Description of Additional Supplementary Data Files

Supplementary Data 1. The Excel file includes Gene-based burden test results based on rare and damaging variants in the discovery stage.

Supplementary Data 2. The Excel file comprises summary of information for 102 PRP patients.

Supplementary Data 3. The Excel file comprises the KEGG signal transduction pathways information of PRP with *KRT3*2 mutations.

Supplementary Data 4. The Excel file includes RNA-seq of formalin-fixed paraffin-embedded (FFPE) from 6 Pityriasis rubra pilaris (PRP) with *KRT32* mutations.

Supplementary Data 5. The Excel file comprises the KEGG signal transduction pathways information of Ker-CT cells overexpression KRT32.

Supplementary Data 6. The Excel file includes RNA-seq of negative control and KRT32-overexpressing Ker-CT cells.

Supplementary Data 7. The Excel file includes KRT32 binding proteins.

Supplementary Data 8. The Excel file includes the KEGG signal transduction pathways information of *Krt32* knockout mice.

Supplementary Data 9. The Excel file includes RNA-seq of *Krt32* knockout and wildtype mice dermal response to TNF stimulation.
