# Supplementary material for: Loss-of-function mutations in Keratin 32 gene disrupt skin immune homeostasis in pityriasis rubra pilaris
Source: Nat Commun. 2024 Jul 24;15:6259. doi: 10.1038/s41467-024-50481-z (PMC11269665; doi:10.1038/s41467-024-50481-z)
Supplement: Supplementary file 4 — Supplementary Data 1-9 [file 41467_2024_50481_MOESM4_ESM.zip › Supplementary Dataset/Description of Additional Supplementary Data files.docx]

**Description** **of Additional Supplementary Data Files**

**Supplementary Data 1**. The Excel file includes Gene-based burden test results based on rare and damaging variants in the discovery stage.

**Supplementary Data 2**. The Excel file comprises summary of information for 102 PRP patients.

**Supplementary Data 3**. The Excel file comprises the KEGG signal transduction pathways information of PRP with *KRT3*2 mutations.

**Supplementary Data 4**. The Excel file includes RNA-seq of formalin-fixed paraffin-embedded (FFPE) from 6 Pityriasis rubra pilaris (PRP) with *KRT32* mutations.

**Supplementary Data 5**. The Excel file comprises the KEGG signal transduction pathways information of Ker-CT cells overexpression KRT32.

**Supplementary Data 6**. The Excel file includes RNA-seq of negative control and KRT32-overexpressing Ker-CT cells.

**Supplementary Data 7**. The Excel file includes KRT32 binding proteins.

**Supplementary Data 8**. The Excel file includes the KEGG signal transduction pathways information of *Krt32* knockout mice.

**Supplementary Data 9**. The Excel file includes RNA-seq of *Krt32* knockout and wildtype mice dermal response to TNF stimulation.
