# Supplementary material for: Loss-of-function mutations in Keratin 32 gene disrupt skin immune homeostasis in pityriasis rubra pilaris
Source: Nat Commun. 2024 Jul 24;15:6259. doi: 10.1038/s41467-024-50481-z (PMC11269665; doi:10.1038/s41467-024-50481-z)
Supplement: Supplementary file 5 — Reporting Summary [file 41467_2024_50481_MOESM5_ESM.pdf]

nature portfolio

Corresponding author(s): Hong Liu, and Furen Zhang

Last updated by author(s): May 5, 2024

Reporting Summary

Nature Portfolio wishes to improve the reproducibility of the work that we publish. This form provides structure for consistency and transparency in reporting. For further information on Nature Portfolio policies, see our Editorial Policies and the Editorial Policy Checklist.

Statistics

For all statistical analyses, confirm that the following items are present in the figure legend, table legend, main text, or Methods section.

n/a	Confirmed	
☐	☒	The exact sample size (*n*) for each experimental group/condition, given as a discrete number and unit of measurement
☐	☒	A statement on whether measurements were taken from distinct samples or whether the same sample was measured repeatedly
☐	☒	The statistical test(s) used AND whether they are one- or two-sided<br>*Only common tests should be described solely by name; describe more complex techniques in the Methods section.*
☐	☒	A description of all covariates tested
☐	☒	A description of any assumptions or corrections, such as tests of normality and adjustment for multiple comparisons
☐	☒	A full description of the statistical parameters including central tendency (e.g. means) or other basic estimates (e.g. regression coefficient) AND variation (e.g. standard deviation) or associated estimates of uncertainty (e.g. confidence intervals)
☐	☒	For null hypothesis testing, the test statistic (e.g. *F*, *t*, *r*) with confidence intervals, effect sizes, degrees of freedom and *P* value noted<br>*Give P values as exact values whenever suitable.*
☒	☐	For Bayesian analysis, information on the choice of priors and Markov chain Monte Carlo settings
☒	☐	For hierarchical and complex designs, identification of the appropriate level for tests and full reporting of outcomes
☐	☒	Estimates of effect sizes (e.g. Cohen's *d*, Pearson's *r*), indicating how they were calculated

Our web collection on statistics for biologists contains articles on many of the points above.

Software and code

Policy information about availability of computer code

Data collection	No software used to collect the data
Data analysis	The AlphaFold program was utilized to obtain a rational three-dimensional structural model of KRT32 and NEMO proteins. The PROCHECK program was used to evaluate and assess the stereochemical quality and accuracy of the protein model through Ramachandran map calculations after refinement. HDDock program was used for molecular docking studies. PYMAL 2.5 was used for visualize the 3D simulated structures of KRT32 and NEMO proteins. GraphPad Prism 8.3 was used for creating graphs and doing statistical analysis. IBM SPSS Statistics 26 was used for statistical analysis. Adobe Photoshop 2022 and illustrator 2022 were used to create figures.

For manuscripts utilizing custom algorithms or software that are central to the research but not yet described in published literature, software must be made available to editors and reviewers. We strongly encourage code deposition in a community repository (e.g. GitHub). See the Nature Portfolio guidelines for submitting code & software for further information.

Data

Policy information about availability of data

All manuscripts must include a data availability statement. This statement should provide the following information, where applicable:

- Accession codes, unique identifiers, or web links for publicly available datasets
- A description of any restrictions on data availability
- For clinical datasets or third party data, please ensure that the statement adheres to our policy

The RNA-seq data from Krt32 knockout and wildtype mice dermal response to TNF stimulation in this study have been deposited in the Genome Sequence Archive

(GSA) database under accession code CRA015401 [https://ngdc.cncb.ac.cn/gsa]. The RNA-seq data of KRT32 overexpressing Ker-CT cell lines, and FFPE samples from 6 PRP with KRT32 mutations in this study have been deposited in the Genome Sequence Archive (GSA) for Human database under accession code HRA006924 and HRA006901 [https://ngdc.cncb.ac.cn/gsa-human] respectively. The WES data of PRP identifies rare mutations in KRT32 in this study have been deposited in the Genome Sequence Archive (GSA) for Human database under accession code HRA005276 [https://ngdc.cncb.ac.cn/gsa-human]. The protein data regarding the interaction of GST-KRT32 identified through Pulldown-MS in this study has been deposited in the ProteomeXchange Consortium under accession code PXD050141 [https://proteomecentral.proteomexchange.org/ui]. The genetic data obtained from participants in this project have been approval by the China Human Genetic Resources Management Office. All data generated or used during the study appear in the submitted article and its Supplementary Information. Uncropped and unprocessed scans of blots have been provided as in the Source data file. Source data are provided with this paper.

Research involving human participants, their data, or biological material

Policy information about studies with human participants or human data. See also policy information about sex, gender (identity/presentation), and sexual orientation and race, ethnicity and racism.

Reporting on sex and gender	Self-reported sex of participants has been reported in the manuscript. We provided detailed messages of gender in supplementary table 3 and 4.
Reporting on race, ethnicity, or other socially relevant groupings	All individuals were of Chinese descent, which has been specified in the manuscript. We collected the age, sex and other clinical information of all samples in Supplementary Table 3 and 4.
Population characteristics	We collected data from 102 PRP patients (66 males and 36 females) and 800 healthy controls (453 males and 347 females), with ages ranging from 1 to 89 years old. Additionally, we obtained skin tissues from 6 PRP patients with KRT32 mutations (3 males and 3 females), 6 PRP patients without KRT32 mutations (3 males and 3 females) and 6 healthy controls (3 males and 3 females), with ages ranging from 33 to 76 years old. All sex and clinical information for the samples, including individuals carrying pathogenic mutations in KRT32, are detailed in Supplementary Tables 3 and 4.
Recruitment	Full descriptions of the recruitment and design of each contributing study can be found in the Methods. There are no any self-selection bias or other biases.
Ethics oversight	Approval for the collection of all case and control samples was granted by the ethical committee of the Shandong Provincial Institute of Dermatology and Venereology.

Note that full information on the approval of the study protocol must also be provided in the manuscript.

Field-specific reporting

Please select the one below that is the best fit for your research. If you are not sure, read the appropriate sections before making your selection.

[x] Life sciences [ ] Behavioural & social sciences [ ] Ecological, evolutionary & environmental sciences

For a reference copy of the document with all sections, see nature.com/documents/nr-reporting-summary-flat.pdf

Life sciences study design

All studies must disclose on these points even when the disclosure is negative.

Sample size	In the discovery stage, whole exome sequencing (WES) was conducted on 58 patients diagnosed with PRP and 364 healthy controls in order to investigate the genetic basis underlying PRP. An additional cohort comprising of 44 PRP patients and 436 healthy controls was included in the validation analysis utilizing Sanger sequencing. For formalin-fixed paraffin-embedded (FFPE) RNA-seq, tissue samples from 6 patients with PRP carrying KRT32 mutations and 6 age-, gender-, and site-matched healthy controls were obtained. For mice skin RNA-seq, tissue from 8 KRT32 KO mice and 8 matched WT mice.
Data exclusions	To identify novel potentially pathogenic genes associated with PRP, a gene burden test was conducted on all 8,397 genes after excluding one case with damaging CARD14 variants.
Replication	During the discovery phase, 58 patients diagnosed with PRP and 364 healthy controls were included. This was followed by validation using an additional 44 PRP patients and 436 healthy controls. Cellular RNA-seq was conducted with three replicate samples. For mouse skin RNA-seq, tissue from 8 KRT32 knockout (KO) mice and 8 matched wild-type (WT) mice was utilized. Immunoblot, immunohistochemistry, and immunohistochemistry experiments were repeated to ensure consistent and reliable results. Refer to the legend for more details.
Randomization	Randomization was no relevant to our study.
Blinding	Blinding during human sample collection was not feasible due to the individuals' disease status.

Reporting for specific materials, systems and methods

We require information from authors about some types of materials, experimental systems and methods used in many studies. Here, indicate whether each material, system or method listed is relevant to your study. If you are not sure if a list item applies to your research, read the appropriate section before selecting a response.

Materials & experimental systems

n/a	Involved in the study
	☒ Antibodies
	☒ Eukaryotic cell lines
☒	Palaeontology and archaeology
	☒ Animals and other organisms
☒	Clinical data
☒	Dual use research of concern
☒	Plants

Methods

n/a	Involved in the study
☒	ChIP-seq
	☒ Flow cytometry
☒	MRI-based neuroimaging

Antibodies

Antibodies used

Antibody (catalog number, Supplier name, clone name, lot number)
anti-Flag (14793, Cell Signaling Technology, D6W5B,)
anti-Flag (8146, Cell Signaling Technology, 9A3)
anti-HA(3724, Cell Signaling Technology, C29F4)
anti-p-IKKa/β (2697, Cell Signaling Technology,16A6)
anti-p-p65 (3033, Cell Signaling Technology,93H1)
anti-p65 (8242, Cell Signaling Technology,D14E12)
anti-NEMO (18474-1-AP, PROTEINTECH)
anti-GAPDH (60004-1-Ig, PROTEINTECH,1E6D9)
anti-KRT32 (H00003882-B01P, Abnova)
anti-KRT32 (H00003882-D01P, Abnova)
anti-TNF (BA0131, Boster)
anti-IL-1β (sc-32294, Santa Cruz, E7-2-hIL1β)
anti-IL-6 (ab9324, Abcam,1.2-2B11-2G10)
anti-IL-8 (27095-1-AP, PROTEINTECH)
anti-Ki67 (A21861, abclonal, ARC5050-01)
anti-IKKα (11930, Cell Signaling Technology,3G12).
anti-NEMO (SC-8032, Santa Cruz,B-3)
anti-Ub antibodies (1ab7780, abcam)
anti-Ub antibody (SC-8017, Santa Cruz,P4D1)
anti-Ub (3936, CST,P4D1)
anti-K48 UB (4289, CST)

Validation

All antibodies are commercially available and their manufactures provided their validation documents. They were validated for WB or IF or IHC.
https://www.cellsignal.cn/products/primary-antibodies/dykddddk-tag-d6w5b-rabbit-mab-binds-to-same-epitope-as-sigma-aldrich-anti-flag-m2-antibody/14793
https://www.cellsignal.cn/products/primary-antibodies/dykddddk-tag-9a3-mouse-mab-binds-to-same-epitope-as-sigma-aldrich-anti-flag-m2-antibody/8146
https://www.cellsignal.cn/products/primary-antibodies/ha-tag-c29f4-rabbit-mab/3724
https://www.cellsignal.com/products/primary-antibodies/phospho-ikka-b-ser176-180-16a6-rabbit-mab/2697
https://www.cellsignal.com/products/primary-antibodies/phospho-nf-kb-p65-ser536-93h1-rabbit-mab/3033
https://www.cellsignal.com/products/primary-antibodies/nf-kb-p65-d14e12-xp-rabbit-mab/8242
https://www.ptgcn.com/products/IKBKG-Antibody-18474-1-AP.htm
https://www.ptgcn.com/products/GAPDH-Antibody-60004-1-Ig.htm
https://www.abnova.com/products/products_detail.asp?Catalog_id=H00003882-B01P
https://www.abnova.com/en-global/product/detail/h00003882-d01p
https://www.boster.com.cn/index/products/productsDetail?goods_sn=BA0131
https://www.scbt.com/p/il-1beta-antibody-e7-2-hilbeta
https://www.abcam.com/products/primary-antibodies/il-6-antibody-12-2b11-2g10-ab9324.html
https://www.ptgcn.com/products/CXCL8-IL8-Antibody-27095-1-AP.htm
https://abclonal.com.cn/catalog/A21861
https://www.cellsignal.cn/products/primary-antibodies/ikka-3g12-mouse-mab/11930
https://www.scbt.com/p/ikkgamma-antibody-b-3
https://www.abcam.com/products/primary-antibodies/ubiquitin-antibody-ab7780.html
https://www.cellsignal.cn/products/primary-antibodies/k48-linkage-specific-polyubiquitin-antibody/4289
https://www.scbt.com/p/ubiquitin-antibody-p4d1
https://www.cellsignal.cn/products/primary-antibodies/ubiquitin-p4d1-mouse-mab/3936

Eukaryotic cell lines

Policy information about cell lines and Sex and Gender in Research

Cell line source(s)	Ker-CT cells were obtained from the American Type Culture Collection, under the catalog number CRL-4048.
Authentication	The cell lines have been authenticated by Short Tandem Repeat (STR) analysis.
Mycoplasma contamination	All the cells used in this study were tested Mycoplasma-negative.
Commonly misidentified lines (See ICLAC register)	No commonly misidentified cell lines were used.

Animals and other research organisms

Policy information about studies involving animals; ARRIVE guidelines recommended for reporting animal research, and Sex and Gender in Research

Laboratory animals	Krt32 wildtye (WT) and knockout (KO) C57BL/6J mice (generated by Cyagen Biosciences, China) female and male mice of 8 weeks were keeped in specific pathogen free conditions with a regular 12h light/12 dark cycle, at approximately 20-25°C and 45-60% humidity.
Wild animals	Homozygous knockout mice krt32(-/-) and their corresponding control littermates krt32(+/+) were generated by breeding of heterozygous krt32(+/-) mice.
Reporting on sex	Sex was not considered in this study.
Field-collected samples	No filed collected samples were used in this study.
Ethics oversight	No filed collected samples were used in this study. Ethics oversight The experiments performed were according to the instructions and permissions of the ethical committee of the Shandong First Medical University.

Note that full information on the approval of the study protocol must also be provided in the manuscript.

Flow Cytometry

Plots

Confirm that:

- [x] The axis labels state the marker and fluorochrome used (e.g. CD4-FITC).
- [x] The axis scales are clearly visible. Include numbers along axes only for bottom left plot of group (a 'group' is an analysis of identical markers).
- [x] All plots are contour plots with outliers or pseudocolor plots.
- [x] A numerical value for number of cells or percentage (with statistics) is provided.

Methodology

Sample preparation	The Ker-CT cells were infected with recombinant lentivirus expressing KRT32 or control recombinant lentivirus for 72 hours, followed by a two-week selection period using hygromycin B. Subsequently, the cells were harvested, fixed, and stained with Flag-labeled antibodies and FITC-labeled antibodies for flow cytometry analysis.
Instrument	BD FACSCanto TM II
Software	Flowjo V10
Cell population abundance	The experiment did not involve post-sort fractions.
Gating strategy	Debris and doublets were excluded first by FSC/SSC gating. The cells infected with recombinant lentivirus, screened with hygromycin B, and performed the same fixation and staining treatments as the experimentao group were considerd as FITC negative cells. Then FITC negative cells were gated in the SSC vs. FITC plot, followed by detection of the negative cells. Cells with FITC fluorescence expression intensity higher than negative cells were considered positive cells.

- [x] Tick this box to confirm that a figure exemplifying the gating strategy is provided in the Supplementary Information.
